# Supplementary material for: Genetic Identification and Technological Potential of Indigenous Lactic Acid Bacteria Isolated from Alheira, a Traditional Portuguese Sausage
Source: Foods. 2024 Feb 16;13(4):598. doi: 10.3390/foods13040598 (PMC10888191; doi:10.3390/foods13040598)
Supplement: Supplementary file 1 [file foods-13-00598-s001.zip › foods-2690507-supplementary.pdf]

Supplementary Table S1. Summary statistics (minimum [Min], first quantile [1st Qu], median, mean, third quantile [3rd Qu], and maximum [Max] values) of phenotypic data from lactic acid bacteria isolated from *alheira*, a Portuguese fermented meat sausage. Legend: ΔpH03, ΔpH06, ΔpH36: pH drop between t=0 h and t=3 h, t=0 h and t=6 h, and t=3 h and t=6 h at 30 °C, respectively; pH6: pH value after 6 h at 30 °C; ProteolyticAct: diameter of proteolytic activity (mm) measure in vitro; LAC: L-lactic acid (g/L). For each pathogen: ID_10C and ID_37C: diameter of inhibition (mm) tested at 10 °C and 37 °C, respectively.

**Species**	**Min.**	**1st Qu.**	**Median**	**Mean**	**3rdQu.**	**Max.**
Enterococcus faecium (n=20)						
Proteolytic Activity (mm)	0.000	0.000	0.000	0.055	0.000	1.110
pH6	5.711	5.756	5.786	5.786	5.812	5.895
ΔpH03	0.244	0.306	0.332	0.331	0.355	0.380
ΔpH06	0.741	0.817	0.856	0.848	0.875	0.916
ΔpH36	0.488	0.501	0.516	0.516	0.529	0.539
LAC (g/L)	0.162	0.237	0.273	0.3253	0.4155	0.5911
ID_10C *L. monocytogenes* (mm)	7.553	11.249	11.486	11.291	11.685	11.940
ID_37C *L. monocytogenes* (mm)	5.995	8.021	8.320	8.196	8.421	9.235
ID_10C *S. aureus* (mm)	3.490	4.638	4.954	4.876	5.215	5.628
ID_37C *S. aureus* (mm)	0.000	0.000	0.000	0.181	0.404	0.765
ID_10C *S.* Typhimurium (mm)	2.808	3.723	4.335	4.266	4.711	6.130
ID_37C *S.* Typhimurium (mm)	0.000	0.000	0.000	0.000	0.000	0.000
Lacticaseibacillus paracasei (n=6)						
Proteolytic Activity (mm)	0.000	2.300	4.256	3.462	4.968	4.995
pH6	5.793	5.824	5.889	6.038	6.398	6.438
ΔpH03	0.057	0.082	0.238	0.197	0.271	0.297
ΔpH06	0.111	0.156	0.738	0.556	0.792	0.799
ΔpH36	0.029	0.099	0.497	0.358	0.499	0.527
LAC (g/L)	0.047	0.055	0.062	0.252	0.644	0.645
ID_10C *L. monocytogenes* (mm)	16.730	17.200	17.440	17.670	18.280	18.710
ID_37C *L. monocytogenes* (mm)	9.182	9.954	10.414	10.447	10.854	11.873
ID_10C *S. aureus* (mm)	7.100	8.109	9.236	9.334	10.405	11.902
ID_37C *S. aureus* (mm)	8.410	8.675	8.869	9.065	9.445	9.992
ID_10C *S.* Typhimurium (mm)	8.648	8.805	9.744	9.750	10.606	10.977

ID_37C *S.* Typhimurium (mm)	9.967	10.949	11.611	11.520	11.922	13.175
Lactiplantibacillus herbarum (n=2)						
Proteolytic Activity (mm)	0.000	0.000	0.410	0.410	0.820	0.820
pH6	6.477	6.477	6.478	6.478	6.478	6.478
ΔpH03	0.106	0.106	0.131	0.131	0.155	0.155
ΔpH06	0.162	0.162	0.188	0.188	0.213	0.213
ΔpH36	0.056	0.056	0.056	0.056	0.057	0.057
LAC (g/L)	0.529	0.529	0.557	0.557	0.585	0.585
ID_10C *L. monocytogenes* (mm)	17.410	18.280	19.140	19.140	20.000	20.860
ID_37C *L. monocytogenes* (mm)	11.400	11.600	11.790	11.790	11.980	12.170
ID_10C *S. aureus* (mm)	10.720	11.010	11.290	11.290	11.580	11.870
ID_37C *S. aureus* (mm)	7.662	7.669	7.675	7.675	7.681	7.688
ID_10C *S.* Typhimurium (mm)	10.940	11.070	11.210	11.210	11.340	11.480
ID_37C *S.* Typhimurium (mm)	9.262	9.779	10.295	10.295	10.811	11.328
Lactiplantibacillus plajomi (n=3)						
Proteolytic Activity (mm)	2.053	2.053	3.335	3.068	3.817	3.817
pH6	5.804	5.804	6.471	6.249	6.471	6.471
ΔpH03	0.155	0.155	0.189	0.224	0.329	0.329
ΔpH06	0.230	0.230	0.239	0.430	0.820	0.820
ΔpH36	0.049	0.049	0.075	0.205	0.490	0.490
LAC (g/L)	0.092	0.092	0.514	0.427	0.676	0.676
ID_10C *L. monocytogenes* (mm)	19.180	19.490	19.800	21.620	22.830	25.860
ID_37C *L. monocytogenes* (mm)	9.375	9.920	10.465	10.282	10.735	11.005
ID_10C *S. aureus* (mm)	8.332	10.932	13.533	11.915	13.706	13.880
ID_37C *S. aureus* (mm)	7.478	7.834	8.190	8.233	8.611	9.033
ID_10C *S.* Typhimurium (mm)	9.375	9.887	10.400	10.809	11.526	12.652
ID_37C *S.* Typhimurium (mm)	9.842	9.893	9.943	10.967	11.529	13.115
Lactiplantibacillus plantarum (n=4)						
Proteolytic Activity (mm)	1.780	2.095	3.315	3.219	4.439	4.468
pH6	5.834	5.856	6.069	6.101	6.314	6.433
ΔpH03	0.117	0.155	0.211	0.200	0.256	0.258

ΔpH06	0.277	0.368	0.574	0.546	0.752	0.758
ΔpH36	0.109	0.238	0.386	0.346	0.494	0.503
LAC (g/L)	0.248	0.332	0.423	0.396	0.487	0.462
ID_10C *L. monocytogenes* (mm)	17.120	18.380	20.170	19.770	21.560	21.640
ID_37C *L. monocytogenes* (mm)	9.127	9.182	9.774	9.778	10.370	10.438
ID_10C *S. aureus* (mm)	9.102	9.770	10.611	10.828	11.669	12.988
ID_37C *S. aureus* (mm)	7.475	7.544	7.798	8.299	8.552	10.125
ID_10C *S.* Typhimurium (mm)	9.310	9.842	10.158	10.085	10.400	10.715
ID_37C *S.* Typhimurium (mm)	10.610	10.930	11.560	11.560	12.190	12.500
Latilactobacillus sakei (n=11)						
Proteolytic Activity (mm)	0.000	0.000	0.000	0.759	0.852	4.287
pH6	6.367	6.387	6.434	6.446	6.511	6.519
ΔpH03	0.077	0.119	0.158	0.176	0.259	0.301
ΔpH06	0.148	0.171	0.231	0.239	0.302	0.339
ΔpH36	0.038	0.042	0.050	0.063	0.077	0.146
LAC (g/L)	0.015	0.148	0.562	0.420	0.620	0.650
ID_10C *L. monocytogenes* (mm)	10.790	12.850	14.100	14.620	16.160	19.660
ID_37C *L. monocytogenes* (mm)	10.070	10.520	11.300	11.350	11.970	13.160
ID_10C *S. aureus* (mm)	5.742	7.656	8.307	9.330	8.908	10.775
ID_37C *S. aureus* (mm)	5.662	6.112	6.310	6.512	6.907	7.545
ID_10C *S.* Typhimurium (mm)	6.787	8.239	9.045	8.854	9.461	11.23
ID_37C *S.* Typhimurium (mm)	7.503	8.406	9.510	9.215	9.887	11.113
Leuconostoc mesenteroides (n=12)						
Proteolytic Activity (mm)	0.000	1.682	3.490	3.436	4.918	8.258
pH6	5.995	6.335	6.385	6.356	6.429	6.449
ΔpH03	0.110	0.131	0.165	0.181	0.226	0.301
ΔpH06	0.215	0.223	0.270	0.308	0.321	0.717
ΔpH36	0.079	0.091	0.099	0.126	0.113	0.416
LAC (g/L)	0.011	0.018	0.025	0.055	0.031	0.21
ID_10C *L. monocytogenes* (mm)	15.090	18.590	19.510	19.550	21.230	23.190
ID_37C *L. monocytogenes* (mm)	9.045	9.440	10.345	10.529	11.592	12.280

ID_10C *S. aureus* (mm)	9.275	9.983	10.643	10.617	11.239	11.965
ID_37C *S. aureus* (mm)	5.255	5.740	6.349	6.373	6.982	7.732
ID_10C *S.* Typhimurium (mm)	9.590	10.280	10.810	10.890	11.390	12.570
ID_37C *S.* Typhimurium (mm)	10.420	12.100	13.050	12.890	13.550	14.670
Pediococcus pentosaceus (n=3)						
Proteolytic Activity (mm)	0.000	0.000	4.735	3.403	5.475	5.475
pH6	6.379	6.379	6.406	6.419	6.474	6.474
ΔpH03	0.140	0.140	0.152	0.172	0.225	0.225
ΔpH06	0.184	0.184	0.225	0.240	0.310	0.310
ΔpH36	0.043	0.043	0.073	0.067	0.084	0.084
LAC (g/L)	0.028	0.028	0.559	0.417	0.663	0.663
ID_10C *L. monocytogenes* (mm)	18.050	19.130	20.210	19.660	20.460	20.710
ID_37C *L. monocytogenes* (mm)	9.078	9.796	10.515	10.105	10.619	10.723
ID_10C *S. aureus* (mm)	7.617	9.189	10.760	10.163	11.436	12.113
ID_37C *S. aureus* (mm)	6.202	6.599	6.995	6.740	7.009	7.022
ID_10C *S.* Typhimurium (mm)	10.420	10.550	10.680	10.660	10.780	10.870
ID_37C *S.* Typhimurium (mm)	9.107	9.981	10.855	10.410	11.061	11.268
Weissella viridescens (n=1)						
Proteolytic Activity (mm)	0.000	0.000	0.000	0.000	0.000	0.000
pH6	6.449	6.449	6.449	6.449	6.449	6.449
ΔpH03	0.135	0.135	0.135	0.135	0.135	0.135
ΔpH06	0.239	0.239	0.239	0.239	0.239	0.239
ΔpH36	0.104	0.104	0.104	0.104	0.104	0.104
LAC (g/L)	0.308	0.308	0.308	0.308	0.308	0.308
ID_10C *L. monocytogenes* (mm)	18.210	18.210	18.210	18.210	18.210	18.210
ID_37C *L. monocytogenes* (mm)	11.100	11.100	11.100	11.100	11.100	11.100
ID_10C *S. aureus* (mm)	11.240	11.240	11.240	11.240	11.240	11.240
ID_37C *S. aureus* (mm)	6.185	6.185	6.185	6.185	6.185	6.185
ID_10C *S.* Typhimurium (mm)	10.430	10.430	10.430	10.430	10.430	10.430
ID_37C *S.* Typhimurium (mm)	9.883	9.883	9.883	9.883	9.883	9.883
